# Hyperbaric oxygen therapy: future prospects in regenerative therapy and anti-aging

**DOI:** 10.3389/fragi.2024.1368982

**Published:** 2024-05-02

**Authors:** Manoj Gupta, Jaishriram Rathored

**Affiliations:** ^1^ Datta Meghe Institute of Medical Sciences, Wardha, India; ^2^ Datta Meghe Institute of Higher Education and Research, Wardha, Maharashtra, India

**Keywords:** hyperbaric medicine, regeneration, aging, senescence, telomere, hyperoxia

## Abstract

Hyperbaric Oxygen Therapy (HBOT) utilizes 100% oxygen at high atmospheric pressure for clinical applications. HBOT has proven to be an effective supplementary treatment for a variety of clinical and pathological disorders. HBOT’s therapeutic results are based on the physiological effects of increased tissue oxygenation, or improved oxygen bioavailability. HBOT’s current indications in illnesses like as wound healing, thermal or radiation burns, and tissue necrosis point to its function in facilitating the regeneration process. Various research has revealed that HBOT plays a function in vascularization, angiogenesis, and collagen production augmentation. Individual regeneration capacity is influenced by both environmental and genetic factors. Furthermore, the regenerating ability of different types of tissues varies, and this ability declines with age. HBOT affects physiological processes at the genetic level by altering gene expression, delaying cell senescence, and assisting in telomere length enhancement. The positive results in a variety of indications, ranging from tissue regeneration to better cognitive function, indicate that it has enormous potential in regenerative and anti-aging therapy.

## Introduction

Hyperbaric oxygen therapy (HBOT) involves the therapeutic utilization of oxygen at a pressure higher than the normal atmospheric pressure (Tibbles and Edelsberg, 1996; Edwards, 2010a). HBOT has been shown to be a useful therapeutic method in a variety of clinical disorders, including wound healing, carbon monoxide (CO) poisoning, respiratory illnesses, and decompression sickness (Edwards, 2010b; Cianci et al., 2013; Levett et al., 2015). A vast number of clinical investigations have shown that increased pressure and increased oxygen bioavailability during HBOT regulates many processes such as angiogenesis, immunological activation, and tissue regeneration (Kuffler, 2011; Grassmann et al., 2015; Memar et al., 2019; Park et al., 2019). However, in most of the therapeutic applications of HBOT, the underlying mechanisms are not well understood and thus, the potential areas of its application are limited to the current knowledge.

Regenerative medicine focuses on either repairing or replenishing damaged tissues through a variety of methods or modifying cellular circumstances to encourage tissues’ natural self-healing abilities. Tissue engineering and stem cell treatment are approaches that involve the transfer of therapeutic cells that restore the structure and function of damaged tissues and stimulate regeneration (Usas and Huard, 2007; Biehl and Russell, 2009). Tissue regeneration is a highly regulated process, with different degrees of regeneration depending on the type of tissue and the cellular milieu. Another strategy for replenishing injured tissues is tissue grafting from autologous or allogeneic sources (Mason and Dunnill, 2009; Berner et al., 2013). Successful tissue grafting requires efficient vascularization of graft to be anastomosed with the host blood vessels (Sanders and Mayou, 1979; Ben-Shaul et al., 2019). Thus, providing a cellular microenvironment to enhance the efficiency of existing regenerative therapies can be of utmost importance.

With the passage of time, ageing is a universal process of progressive deterioration of physiological processes and loss of effectiveness of all critical organs. The ageing process is a complicated one, and several theories have been proposed to better explain it. One of the most important aspects of ageing is the loss of the body’s ability to maintain homeostasis. As a result, the regeneration and repair process slows down, affecting key processes such as heart function, lung capacity, renal function, motor function, and neural function (Esler et al., 1995; Ossowska et al., 2001; Denic et al., 2016; Tudorache et al., 2017; Fajemiroye et al., 2018). Aging causes an increase in the rate of mutation, oxidative damage due to a decrease in mitochondrial activity, connective tissue loss, and muscle atrophy (Borst and Lowenthal, 1997; Bailey, 2001; Short et al., 2005). Since oxygen is a vital regulator of several aspects of cellular activities, an individual’s metabolic rate is a significant factor of cellular health. Variations in oxygen tension in the body can have an impact on cellular respiration, which is linked to cellular ageing. As a result, clinical modification of oxygen tension in an individual may have therapeutic implications in terms of slowing down the ageing process.

HBOT has been used in therapeutic applications such as necrotic injuries, thermal and radiation burns, and tissue regeneration and repair (Villanueva et al., 2004; Girnius et al., 2006; Bennett et al., 2016). HBOT’s physiological effects show that it has a role in stem cell dynamics and other tissue regeneration processes like angiogenesis and vascularization (Sanders and Mayou, 1979; Grassmann et al., 2015). HBOT causes hyperoxic conditions, which activate a series of signalling pathways and cause the release of signalling molecules and growth factors. HBOT treatment normally entails numerous sessions, putting the patient to a cycle of increased and normal oxygen levels. Through the activation of a feedback loop, the transformation of hyperoxic/hypoxic state allows for the triggering of secondary effects. Due to a wide range of treatment protocols and a lack of understanding of pressure effects on human physiology, the mechanism of therapeutic outcomes of HBOT is poorly known. HBOT, on the other hand, has been shown to boost healing conditions and regeneration capacity. In this review, we have documented the crucial role of HBOT as an adjuvant treatment, particularly in encouraging tissue regeneration, as well as its probable implications on the consequences of cellular ageing.

## Theory and concept of biological aging

Biological ageing is an intriguing phenomenon in which physiological activities gradually cease over time. The most essential characteristic of ageing is decreased capacity of organ systems, which affects pulmonary functions, cardiac functions, renal functions, and musculoskeletal system elasticity, among other things (Whitbourne, 2012). The interplay of many factors reduces the body’s signalling pathways, energy transduction machinery, anti-oxidant ability, and cellular regeneration capacity on a molecular and cellular level (Cao et al., 2004; Navarro and Boveris, 2007; Nedić et al., 2013; Sousounis et al., 2014). Several theories have been postulated to understand the phenomenon of aging but a single theory may not be exclusive in understanding the concept of aging.

There are two basic theories of biological ageing (programmed theory and damage and error theory), each of which has multiple subcategories based on the mechanisms that cause degradation. Aging, like all biological events in an organism’s life cycle, is a process based on a biological clock, according to the programmed theory of ageing. As a result, it is a predefined or programmed process that is initiated by internal causes. The three aspects of programmed theory are programmed longevity, endocrine theory, and immunological theory of ageing (Barzilai et al., 2010; Jin, 2010; Fulop et al., 2014; Longo, 2019). According to the programmed longevity theory, the ageing process is dependent on the “ON” and “OFF” of specific genes, which signals the commencement of senescence in an individual. The endocrine hypothesis of ageing proposes that hormones govern ageing, with the evolutionary conserved insulin/IGF-1 signalling pathway playing a key role in hormonal ageing regulation (Anisimov and Bartke, 2013; Tullet et al., 2014). The immunological hypothesis of ageing focuses on an individual’s immune deterioration with time, which increases susceptibility to infections and disorders. As the immune system deteriorates, the homeostatic balance is disrupted, resulting in inflammation, cellular changes, and a loss of muscle flexibility, among other consequences (Ponnappan and Ponnappan, 2011). Damage and error theory, in contrast to the planned explanation of ageing, emphasises the effect of external variables on an individual, which eventually leads to a cumulative negative effect on the body. Wear and tear theory, rate of living theory, cross-linking theory, free radicals’ theory, and somatic DNA damage theory are the five subcategories of damage and error theory. According to wear and tear theory, the human body, like a mechanical equipment, experiences steady wear and tear over time (Sattaur et al., 2020). The rate of living theory is based on an individual’s metabolism, with a faster metabolic rate leading to more oxidative damage and eventually a senescent condition (Brys et al., 2007). Cross-linking theory relies on the fact that protein cross linking occurs over time which causes their toxic accumulation and thus results in damage to healthy cells and tissues (Nagy and Nagy, 1980). According to the free radical theory of ageing, the amount of free radicals in the body increases as anti-oxidant capacity decreases. The production of free radicals alters the working condition of macromolecules such as proteins, lipids, and DNA, impairing an individual’s ability to maintain homeostasis (Pomatto and Davies, 2018). The somatic DNA damage theory connects the ageing process to the build up of DNA damage over time. Although cells have a highly robust DNA repair system to guarantee genetic integrity, genetic mutations accumulate with age, causing negative impacts on an individual’s proper functioning of life-sustaining functions (Ribezzo et al., 2016; Horvath and Raj, 2018). Additionally, as telomere length decreases due to telomerase activity restrictions, DNA integrity is jeopardised throughout succeeding cell division cycles. The loss of telomeres over time results in an inability to maintain cell viability, which leads to tissue deterioration (Horvath and Raj, 2018).

It is noteworthy that a single ageing theory may not be sufficient to comprehend the complicated process of ageing, and that the end result is more likely a cumulative impact of all the elements that contribute to aging’s negative effects. While it may not be feasible to reverse the consequences of ageing, focusing on the elements that accelerate the ageing process is surely useful. Therapeutics for ageing are intended to target these causal variables in order to slow down the ageing process. Because oxygen is such a crucial role in determining an individual’s metabolic state, manipulating the oxygenation state can aid in understanding and managing the metabolic variables that cause ageing.

## Physiological and metabolic consequences of aging

Aging is associated with numerous physiological changes all over different organ system due to their reduced capacity and deterioration. The majority of the alterations are irreversible and appear in the fourth or fifth decade of life. The rate of deterioration differs from one person to the next, based on genetics, eating habits, environmental factors, and job characteristics (Finch and Tanzi, 1997; Jex et al., 2007; Vierkötter and Krutmann, 2012; Soultoukis and Partridge, 2016). Unless therapeutic intervention is used, there is a cumulative effect of age-related physiological changes as people get older. It is controversial whether age-related changes are caused by external factors such as wear and tear or are more driven by intrinsic variables such as genetic composition. However, it is more likely that ageing is the result of a combination of internal and external processes.

The normal ageing process is marked by a loss of bone and muscle mass, which increases the risk of fractures and lowers overall quality of life. Individuals’ physical mobility is harmed as skeletal muscle strength deteriorates. The total capacity of muscles is diminished due to the gradual degradation of organelles involved in protein metabolism inside the sarcoplasm. As a result, the size of muscle cells and the number of muscular fibres decrease dramatically (Navarro et al., 2001). Apart from that, the cartilages are subjected to wear and tear, resulting in stiffness and pain in the joints. The majority of people over the age of 50 experience skeletal degradation in the form of mild to severe osteoporosis. Due to hormonal changes connected with menopause, women are more prone to osteoporosis than males (Weinans, 1999). Epidermal atrophy is caused by the loss of skin suppleness as people get older. Collagen and elastin fibres stiffen and calcify, causing wrinkles in the skin (Bhattacharyya and Thomas, 2004). Other non-skeletal muscles, such as heart and respiratory muscles, begin to deteriorate as people get older. People in their later years are more likely to develop heart problems such as arteriosclerosis and coronary artery disease. There is also a stiffening of arteries and calcification of elastic fibres in cardiac muscles and myocardium, which decreases overall cardiac output (Dai and Rabinovitch, 2009). As lung elasticity declines, the respiratory system becomes weaker, resulting in a reduction in vital capacity and arterial oxygen pressure (Sharma and Goodwin, 2006). The immune system is also depressed in older adults, rendering them more prone to illnesses. This is due to a combination of mucociliary insufficiency, which allows for a vulnerable mode of infection, and a general deterioration in the humoral immune system (Akha, 2018). As the total number of glomeruli declines with age, the renal system becomes weaker. As a result, the renal filtration rate decreases, while secondary metabolite levels such as creatinine and urea rise. However, because the rate of creatinine production is likewise slowed, there are lesser variations in creatinine levels. Renal tubule resorption capacity is similarly diminished, resulting in the occurrence of glycosuria (Baylis, 2005; Stähli et al., 2018). Similarly, in old age, the digestive and excretory systems are impacted, with regular disorders such as gastritis, indigestion, decreased intestinal motility, and sphincter muscle weakness (Chernoff, 2014). The loss of hormone-producing cells, such as insulin-producing beta cells in the pancreas and melatonin-producing pinealocytes in the pineal gland, causes the endocrine system to become inefficient. Hormonal imbalances can cause a wide range of disorders, from sleeplessness to diabetes (Jonas et al., 2015; Stucker et al., 2021). Protein amyloids build up in a variety of places, causing degenerative diseases. Deposition of harmful protein aggregates in neuronal cells causes age-related neurodegenerative diseases such as Alzheimer’s disease and Parkinson’s disease, which damage cognitive ability and memory loss (Espay et al., 2019; Sonawane et al., 2021). The deposition of amyloids begins at a young age in many diseases, but the symptoms do not appear until after significant neurodegeneration has already occurred. While most physiological changes linked with ageing are irreversible, having a previous understanding of a person’s clinical status can help to slow down the consequences to a significant degree.

## Aging and regeneration

The ability of an organism to regenerate differs from species to species, as it is developmentally governed by phylogenetic diversity during the course of an organism’s life. The regenerative potential of an organism is often larger throughout its early life stages, and it gets constrained as it ages in organ or tissue specific ways (Poss, 2010). The ability of an organism to regenerate differs from species to species, as it is developmentally governed by phylogenetic diversity during the course of an organism’s life. The regenerative potential of an organism is often larger throughout its early life stages, and it gets constrained as it ages in organ or tissue specific ways (Zhao et al., 2016; Macchi and Sadler, 2020). However, these changes become less prominent once the organism is fully developed physiologically.

In adults, the regeneration process is activated upon cellular injury or insult resulting in the release of systemic factors which acts locally (Bijlsma et al., 1984; Vo et al., 2012). The regenerative response differs by tissue type, but it is not entirely dependent on it. The goal of modern regenerative biology is to uncover the organ- and tissue-specific components that initiate and regulate the regeneration process. The age-related reduction in regenerative capacity can be partially countered by increasing or providing these regenerative elements (Ruckh et al., 2012). In this regard, cellular health and the impact of the environment on an individual’s general physiology are significant factors to consider. As a result, it is possible to conclude that the regeneration rate of individuals belonging to the same species may differ. Because of the increased presence of certain growth factors involved in tissue regeneration, younger people have a higher regenerative ability than older people. It has been discovered that serum from a young animal can induce muscle regeneration in an older animal’s muscles (Conboy et al., 2005). Thus, the systemic factors play an important role in the regenerative capability in the older individuals.

## Hyperbaric oxygen therapy at a glance

Hyperbaric oxygen therapy has become a popular treatment option for a range of medical disorders. HBOT was first used to treat pathophysiological disorders in divers and mine workers, but it has now been expanded to include effective treatment of wounds, thermal or radiation burns, and CO poisoning (Krishnamurti, 2019). HBOT is also used to treat gas embolism, necrotizing soft tissue infections, anaemia, and gas gangrene, among other conditions (Reis et al., 2003; Van Meter, 2005; Gupta, 2010). HBOT is a treatment that includes exposing patients to high-purity oxygen at a pressure higher than normal atmospheric pressure for a set period of time. HBOT treatment involves a series of short-interval sessions based on the patient’s clinical condition and reaction to the treatment. HBOT sessions take place in specialised hyperbaric chambers that can accommodate one or more patients at a time, depending on the chamber type (Jain, 2017). Most HBOT treatments are performed at pressures ranging from 1.4 to 3 atmospheres absolute (ATA), with durations ranging from 30 to 2 h.

The partial pressure of oxygen in arterial blood is 75–100 mmHg under normobaric conditions (normal atmospheric pressure of 1 ATA). HBOT exposes the patient’s body to a larger fractional oxygen content, increasing oxygen bioavailability throughout the body. The therapeutic effect of oxygen on the preservation of an oxidative environment and its impact on physiological functioning has been extensively researched. The multiple effects of higher oxygen bioavailability are widely utilized in treatment of 14 different clinical conditions (Lam et al., 2017). The pressure effect on human physiology can also be ascribed to HBOT’s therapeutic effects. The physiological make-up of people living in higher altitudes with lower atmospheric pressure, as well as people who are constantly exposed to higher atmospheric pressure, such as deep sea divers, demonstrates that the human body is highly sensitive to pressure changes in its environment (Tetzlaff et al., 2001; Wyatt, 2014). When compared to those who are used to normal atmospheric pressure, these people have significantly lower respiratory rates, blood oxygen carrying capacity, haemoglobin concentration, and heart rates. The vascular and neurological system components are sensitive to changes in surrounding pressure and respond by adjusting physiological activities such as lowering heart rate, boosting vasoconstriction, increasing blood flow to central circulation, and releasing stored blood cells from the spleen. Apart from that, higher oxygen pressure causes the release of regulatory molecules that modulate numerous processes such as immunological response, inflammatory response, collagen formation, and anti-oxidant functions in air-filled cavities in the body as well as tissues with higher oxygen demand (Buras et al., 2000; Boykin and Baylis, 2007; Sander et al., 2009; Tal et al., 2017a; Huang et al., 2020). The virtue of oxygen in modulation of these functions enables its use in clinical conditions which requires rapid tissue regeneration and healing.

## Effect of HBOT on aging and cellular regeneration

HBOT has a long history of therapeutic use in a variety of illnesses, but the results of hyperoxic exposure under hyperbaric settings have pointed to its promise in regenerative and anti-aging medicine. In adults, cellular regeneration is hampered by two factors: cellular senescence and telomere shortening (Bernadotte et al., 2016). The senescent cells are destined for programmed cell death as they do not re-enter subsequent cell cycle and are cleared by autophagy. The rate of cellular senescence increases with age as the accumulation of genetic mutation and telomere shortening renders DNA damage to numerous cells, thereby arresting their further propagation (Morgan et al., 2013). There is either increased accumulation of senescent cells or decreased clearance due to weakened immune system in aging individual. Thus, the accumulation of senescent cells further contributes to the process of aging. HBOT has been shown to modulate both cell senescence and telomere shortening indicating its effectiveness in slowing the process of aging.

**FIGURE 1:**
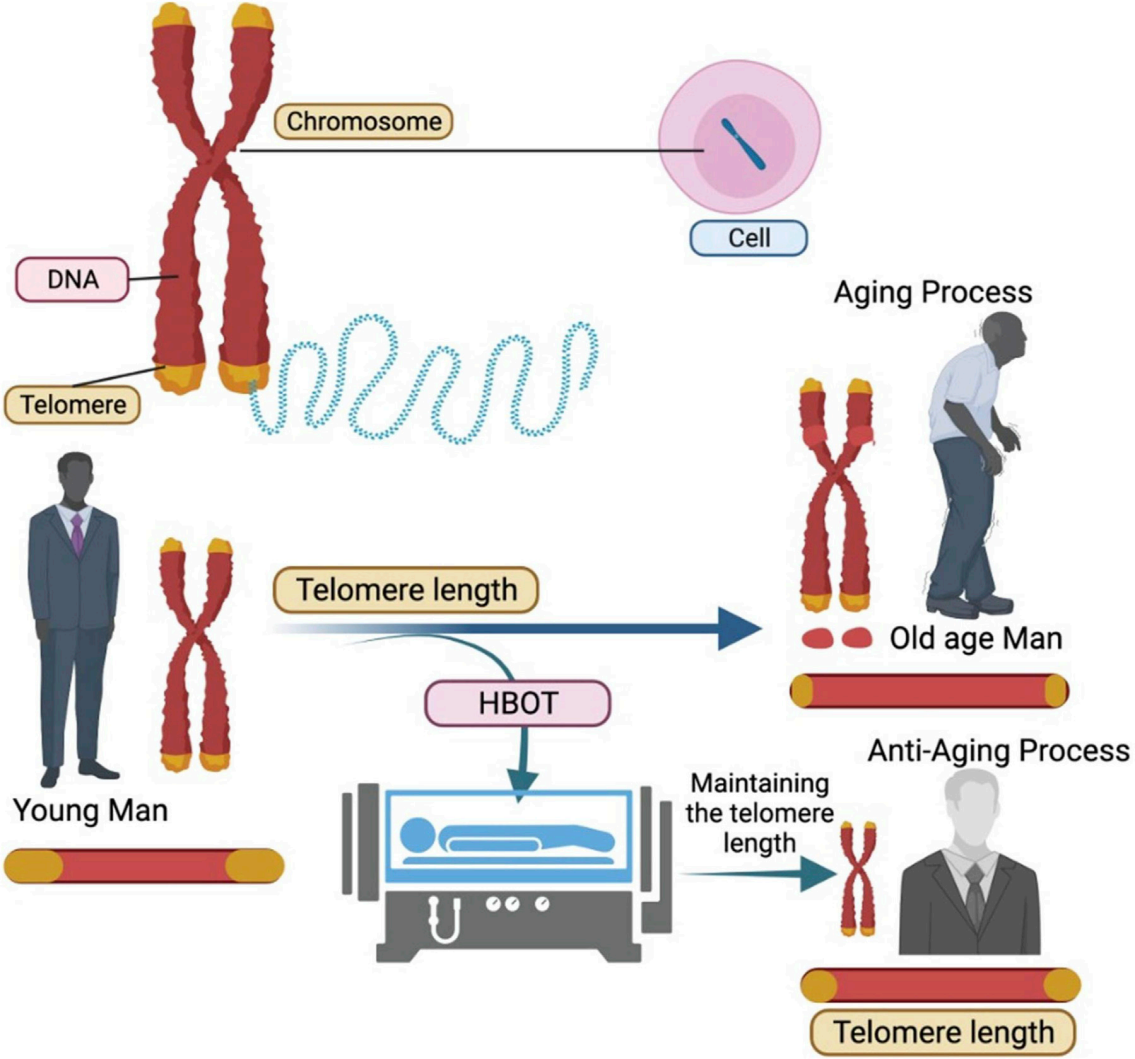
Hyperbaric Oxygen Therapy (HBOT) aids in the maintenance of telomere length resulting in slowing down of aging process.

Telomeres are DNA segments found at the end of chromosomal DNA that include protein-associated repeating non-coding sequences ranging from 4 to 15 kilobases in length (Puhlmann et al., 2019). Telomeres safeguard chromosomal DNA from gradual degradation while also maintaining genetic integrity. After each cell division cycle, the length of the telomeres shortens. Telomerase is a riboenzyme that ensures that telomeres are replenished after each cell division. Telomerase is expressed continuously in cells that cycle often, such as germ cells and epidermal cells. However, because most somatic cells lack telomerase, the length of their telomeres shortens over time, leading to senescence once the telomeres reach a threshold length. Telomeres shorten at a rate of 20–40 bases every year, and as people get older, they accumulate a high number of senescent cells, which adds to the ageing process (Starkweather et al., 2014; Åström et al., 2019). Extrinsic variables can influence telomere shortening rates, such as smoking and vitamin insufficiency, but a balanced lifestyle with correct nutrition, exercise, and certain pharmacological therapies can reduce telomere shortening rates. Because of the harmful effects of reactive oxygen species (ROS) and free radicals, oxidative stress is one of the key mechanisms that drive telomere shortening (Epel et al., 2004; Zhu et al., 2019). Thus, treatment therapies that reduce the effects of reactive oxygen species (ROS) and free radicals may help to protect telomeres. HBOT was investigated for its influence on telomere shortening rates in peripheral blood mononuclear cells (PBMCs), resulting in a 20 percent increase in telomeric length in an ageing population of blood cells. The effect of HBOT on telomere length was most noticeable in B cells. Furthermore, when cells were exposed to HBOT, the number of senescent cells dropped by 10%–37% (Hachmo et al., 2020a). HBOT had the greatest impact on the senescent T-helper cell population. The occasional hyperoxic exposures to cells are responsible for the reduced rates of telomere shortening, increased telomere length, and increased cellular lifetime (Figure 1). According to a previous study, 6 months of aerobic endurance exercise might enhance telomere length by up to 5% (Werner et al., 2019). Aerobic training causes a progressive increase in the oxygen demand of tissues, which is aided by interval exercise. HBOT’s effects are similar to this, with occasional hyperoxia causing a phenomenon known as hyperoxic-hypoxic paradox (91). Hyperoxic-hypoxic paradox has been reported in previous therapeutic uses of oxygen and the effect has been extensively discussed in literature (Balestra and Kot, 2021; Balestra et al., 2023). Hyperoxic-normoxic exposure has been shown to exhibit hematopoietic functions and results in the production of erythropoietin and increased levels of haemoglobin (Salvagno et al., 2022). Intermittent conditions of oxidative stress can arise during situations like physical exercise which can generate the similar effect in the form of eliciting antioxidant protection and erythropoietin production (Revelli et al., 2013; Fratantonio et al., 2021). Significant increase in erythropoietin levels has also been observed in a study involving normobaric oxygen administration in healthy individuals suggesting normobaric paradox (Balestra et al., 2006). The effect is also evident on the transcriptional levels by stabilization of hypoxia inducible factor-1α (HIF-1α), nuclear factor (erythroid-derived 2)-like 2 (NRF2) and nuclear factor kappa-light-chain-enhancer of activated B cells (NF-κB) (Chandel et al., 2000; Fratantonio et al., 2021). The modulation in the telomere transcriptional regulation via HIF-1α and NRF1 can also be attributed to hyperoxic-hypoxic paradox (Diman et al., 2016; Balan et al., 2018; Hachmo et al., 2020b). During HBOT, initial hyperoxic circumstances cause the creation of reactive oxygen species (ROS), which leads to the overexpression of antioxidant genes such as superoxide dismutase (SOD), glutathione reductase (GRx), and glutathione peroxidase (GPx) (Iorio, 2006; Hadanny and Efrati, 2020). The level of ROS drops rapidly during the periods between HBOT sessions, when normobaric conditions predominate, due to their short half-life and the antioxidant impact. Antioxidants, on the other hand, have a longer half-life and continue to be active even when ROS levels return to normal. The consecutive HBOT sessions promotes the higher activity of antioxidants while inducing an initial rise in ROS production. The overall result of repeated HBOT exposures is the generation of a protective cellular environment with minimal oxidative damage. The treatment conditions for utilizing HBOT to generate anti-oxidant effects has been found to be varied in different studies (Balestra et al., 2022; de Wolde et al., 2022; Leveque et al., 2023; MacLaughlin et al., 2023). Leveque et al. (2023), reported comparable levels of ROS, upon 30 min of HBOT under 1.4 ATA and 2.5 ATA of 100% oxygen which peaks after 2 h of treatment. However, another study has shown no significant changes in the plasma ROS levels after 75 min of HBOT at 2.5 ATA of 100% oxygen (de Wolde et al., 2022; Leveque et al., 2023). Thus, the course of treatment in using HBOT depends mostly on the symptomatic factors and initial response to the treatment.

There are a number of different processes that contribute to cellular longevity and the prevention of cell senescence. Hyperoxic environment induces expression of various other genes such as hypoxia induced factors (HIF). HIF induction is related to the regenerative effects of HBOT as it promotes vascularization and angiogenesis through activation of vascular endothelial growth factor (VEGF) (Forsythe et al., 1996; Lando et al., 2003). In traumatic brain injury (TBI), the effect of HBOT on angiogenesis and nerve fibre development has been examined. TBI patients demonstrated improvement in neurocognitive ability, implying that HBOT improved brain microstructure (Tal et al., 2017b). HBOT’s effects on promoting neuroplasticity and cellular healing have also been shown in animal research. HBOT also induce neurogenesis of endogenous neural stems cells suggesting its beneficial role against neurodegeneration. According to Hu et al. (2014), the regenerative benefits of HBOT are mediated through the ROS/HIF-1/-catenin pathway. HBOT was found to boost stem cell mobilisation, cell proliferation, and angiogenesis in a diabetic mouse model, implying that its regenerative potential (Peña-Villalobos et al., 2018). The regenerative effects of HBOT are evident in wound healing and promotion of angiogenesis but further studies are required to understand the underlying mechanisms of regeneration and to better explore the possibilities of HBOT in regenerative medicine.

## Future prospects of HBOT as a regenerative medicine

**FIGURE 2:**
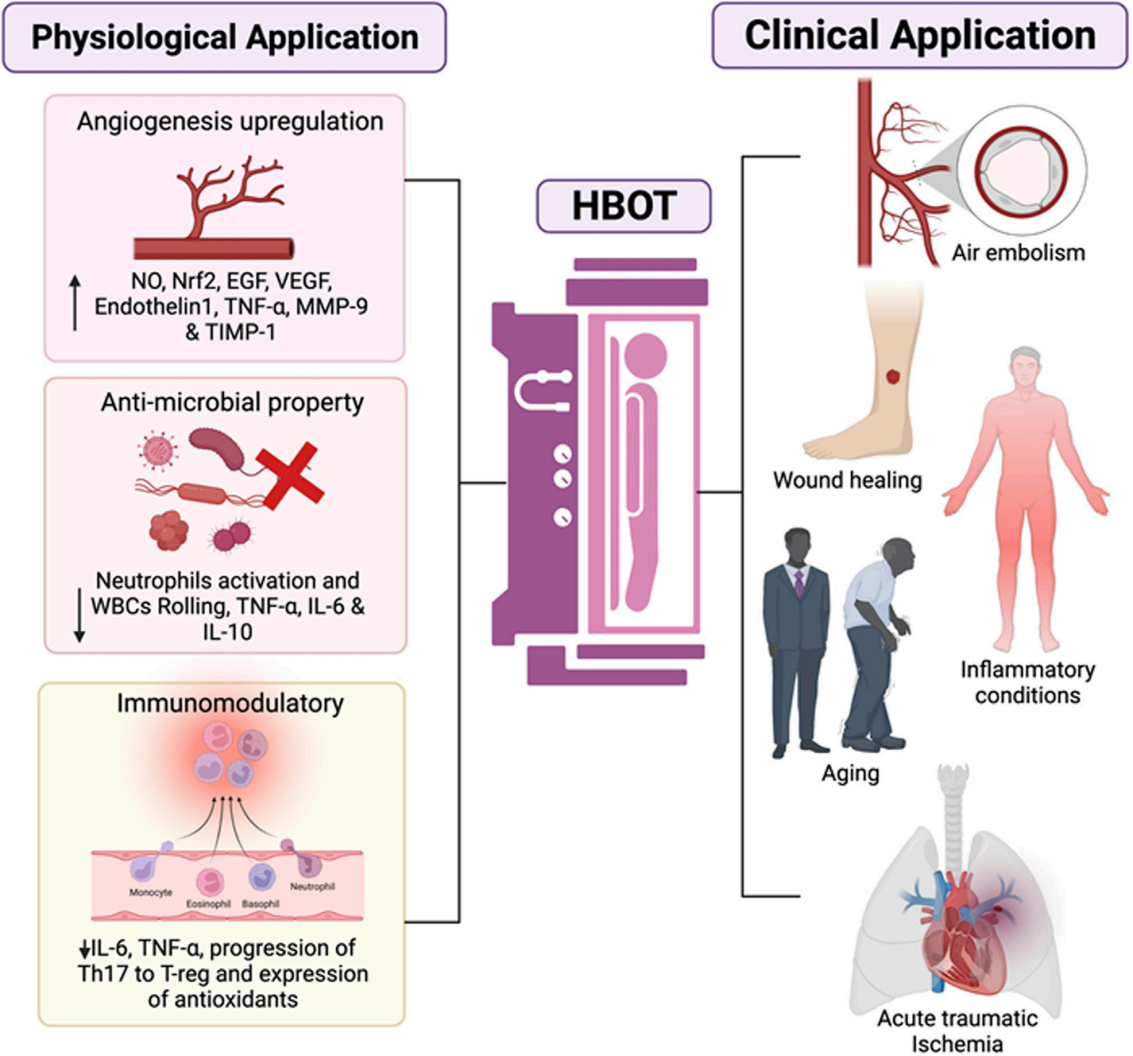
The representative schematic diagram shows the various properties which are influenced by HBOT and their clinical application. For starters, it can promote angiogenesis by increasing NO production, which leads to an increase in Nrf2 and growth factors such as epidermal growth factor (EGF), vascular endothelial growth factor (VEGF), and various associated factors will all be upregulated. Second, antimicrobial activity is visible due to bacterial killing by O_2_, which removes biofilm and reduces white blood cell (WBC) rolling and neutrophil recruitment, promoting the downregulation of proinflammatory cytokines. Downregulation of the transcriptional factors involving a proinflammatory response switch off (IL-6) and a polarisation from Th17 lymphocytes to Treg, is observed to have immunomodulatory properties. Overall, the HBOT application shows clear signs for endorsement of mainly wound healing and infections, primary emergencies (air embolism), and therapeutic interventions (comprising COVID-19, cancer, inflammatory conditions or ageing among others).

HBOT has been tested and approved for application in 14 different clinical conditions (Lam et al., 2017). HBOT works on the mechanism of effects of hyperoxic conditions and increased pressure, which acts on cellular levels and affects the epigenetic regulation of many critical genes. It is estimated that the combined effect of hyperoxic and hyperbaric conditions can alter the gene expression of nearly 40% of protein coding genes (Hadanny et al., 2021). The overall effect of this epigenetic modulation results in physiological consequences in the form of anti-inflammatory effect, anti-apoptotic and regenerative functions. HBOT also exert its effect by modulating the functions of oxygen sensitive gated ion channels and pressure sensitive channels on cellular and mitochondrial membranes. However, there is limited knowledge regarding the functions of these channels with respect to cellular regeneration and general cellular health. The oxygen content at normal atmospheric pressure is 20.9%. Under hyperbaric and hyperoxic conditions, the overall oxygen supply to all the tissues enhances considerably. The intermittent HBOT treatment cycles create hyperoxic-hypoxic paradox, *i.e.*, the body experiences a state of hypoxia without its hazardous effects and induces protective mechanisms in response to hypoxia. The fluctuations in tissue oxygen levels initiate a regenerative mechanism and direct the oxygen supply to the tissues where it is most needed. The extra oxygen supply is utilized in the process of healing, tissue repair, rebuilding and regeneration. The effect is especially beneficial for older individuals as the overall oxygen carrying capacity and regenerative capacity diminishes with age and HBOT helps to compensate for this apparent lack of oxygen in vital processes of healing and regeneration. HBOT provides up to 10–15 times the normal oxygen requirement of body. The hyperoxic environment is beneficial in elevating the natural immune response as the immune cells involved in phagocytosis are dependent on oxygen for their function and gets triggered in hyperoxic conditions to protect against infections. The availability of oxygen is critical in the formation of extracellular matrix responsible for strengthening of tissues. As aging progresses, there is a decline or deterioration of ground material which forms the structural integrity of tissues such as bones, tendons, ligaments, muscles, and skin. Collagen is a major component of these tissues which provides them strength and flexibility. HBOT has been found to accelerate the process of collagen synthesis and shifts the equilibrium towards regeneration instead of degeneration (André-Lévigne et al., 2016). The abundance of oxygen supply aids in the process of repair and slows down the signs of aging. HBOT has been shown to have beneficial effects on the cardiac functions, respiratory functions, and liver functions. The regenerative effect of HBOT has been studied on liver where it was found to enhance liver regeneration after hepatectomy. Symptomatic improvement in urological conditions such as fibrosis, inflammation and submucosal oedema of bladder walls has been reported after treatment course of HBOT for 12 months (Capelli-Schellpfeffer and Gerber, 1999). HBOT showed a marked improvement in cognitive functions and neuronal health which usually declines with aging. The multiple effects of HBOT on a myriad of physiological functions prove its great potency in therapeutics (Figure 2). The regenerative effects of HBOT are well evident in several clinical studies but require extensive research to better understand the mechanisms and to utilize HBOT in a more effective way. While HBOT is proposed to be as an effective measure to slow down age progression, its utilization is limited by the presented contraindications in patients. The occurrence of certain side-effects such as impaired vision, pulmonary complications and sinus damage also limits the use of HBOT as anti-ageing therapy. Furthermore, the anti-ageing effect of HBOT may also be dependent on the genetic constitution of individual, endocrine health and dietary habits; thus, showing possible non-uniformity in anti-ageing effect. Overall, HBOT has shown promising results in regenerative therapy and exhibits anti-aging effect through various mechanisms. The potential of HBOT lies in its ability to modulate multiple functions with minimal side-effects which can be powerful tool to target harmful effects of aging and improve overall health of an individual.
